# Judges’ evaluation reliability changes between identifiable and anonymous performance of hip-hop dance movements

**DOI:** 10.1371/journal.pone.0245861

**Published:** 2021-01-25

**Authors:** Nahoko Sato, Luke S. Hopper

**Affiliations:** 1 Department of Physical Therapy, Faculty of Rehabilitation Science, Nagoya Gakuin University, Nagoya, Aichi, Japan; 2 Western Australian Academy of Performing Arts, Edith Cowan University, Perth, WA, Australia; Jyvaskylan Yliopisto, FINLAND

## Abstract

Hip-hop competitions are performed across the world. In the recent inclusion in the 2018 Youth Olympic Games, the assessment of hip-hop performance is undertaken by a panel of judges. The purpose of this study was to determine the reliability of different visualisation tools utilised in the assessment of the hip-hop dance movements. Ten dancers performed basic rhythmic hip-hop movements which were captured using a motion capture system and video camera. Humanoid and stick figure animations of the dancers’ movements were created from the motion capture data. Ten judges then assessed 20 dance trials through observation using three different visualisation tools on a computer display, each of which provided different representations of a given hip-hop performance: (1) the actual video of the dancers; (2) an anonymous stick figure animation; (3) an anonymous humanoid animation. Judges were not informed that they were repeating an assessment of the performances across the three visualisation tools. The humanoid animation demonstrated the highest inter-class correlation coefficients among the three methods. Despite the stick figure animation demonstrating moderate to high reliability, both the humanoid animation and the video demonstrated very high reliability in the intra-class correlation coefficient. It is recommended that further research is undertaken exploring the use of humanoid animation as a formative assessment tool in the evaluation of hip-hop dance and the evolution of hip-hop into a respected artistic athletic discipline.

## Introduction

Hip-hop dance competitions are performed all over the world. Over 4000 dancers, from over 50 countries participated in the 2019 World Hip Hop Dance Championship [1]. Break dance, a type of hip-hop dance, became an official event in the 2018 Youth Olympic Games [2]. Establishing reliable means of assessing hip-hop performance is essential as hip-hop dance becomes a formalised and internationally competitive entity.

Despite its growing popularity, no systematic method of evaluating the performance of hip-hop dance has been developed and judges’ subjective evaluations of hip-hop dance are potentially unreliable. In general hip-hop dance competitions [1], judges evaluate dancers’ performances on a 10 point scale in several categories without any criteria. A new judging system was developed only for the 2018 Youth Olympic Games. The judging panel consisted of five judges assessing hip-hop performances in real time. The judges assessed dancer’s performance using six categories as shown in Table 1 [2]. All events consisted battles between 2 dancers or 2 teams. A slide bar system was used to determine which dancer or team was better in each category and final scores were calculated. The evaluation criteria of each of the categories was subjective and yet assumed the judges personal evaluations were reliable.

**Table 1:** Six categories which was used to evaluate dance performance in the 2018 Youth Olympic Games.

Domain	Category	Key qualities that judges should be paying attention to as evaluate dancers	Percentage of weighting for final score
Physical Quality: represents the qualities related to the body	Technique	Aptitude & Athleticism, Dexterity-Fine Motor Control, Pushing the Body to the Limit, Balance, Strength & Endurance, Flexibility, Dynamics, Spatial Control	20.0%
	Variety	Dimension, Empathetic Approach	13.3%
Interpretative Quality: represents the qualities related to the Soul	Performativity	Composition & Narrative, The Element of Surprise, Emotional Engagement, Authenticity	20.0%
	Musicality	Coherence, Accenting, Syncopation, Texture, Phrasing	13.3%
Artistic Quality: represents the qualities related to the Mind	Creativity	Progression of the Fundamentals of Foundation	20.0%
	Personality	Stage Presence & Charisma, Individuality & Character	13.3%

In other artistic sporting competitions, objective judging systems have been developed and scrutinised. For example in figure skating, the performances of the contestants are graded according to the difficulty levels of the techniques and the completion level, and the superiority or inferiority of a performance is decided by common objectivity [3]. In artistic gymnastics, scores in each technique are defined by kinematic criteria in “the Code of Points” and many attempts have been made to assess reliability of judging [4–6]. In hip-hop dance, it is considered that kinematic criteria should be defined to support the evolution of hip-hop dance towards a respected artistic competition.

Hip-hop judges’ evaluations are likely based upon multiple aesthetic factors in addition to dancers’ movements such as clothes, facial expressions [7], physical features and body shapes of the performer [8, 9]. In order to extract kinematic elements from hip-hop dance performance evaluations, these confounding aesthetic factors should be anonymised. People are able to distinguish gender of a walking person by observing stick figure animation [10] and this method can be applied for sporting movements [11, 12]. It is assumed that stick figure animation may be able to anonymise aesthetic factors and people are able to gain much perceptual information from that. Sato et al. [13] attempted to assess judges evaluations of hip-hop performances by asking judges to observe dancer routines based on stick figure animations. This approach removed biases of clothes, facial attractiveness, physical features, and body shapes. Although judges from Sato et al. [13] were able to extract kinematic parameters from the hip-hop movements, the judges’ evaluations demonstrated poor reliability. Although people are able to identify movement by observing stick figure animation, it is assumed that it might not provide sufficient information to evaluate expert dance performance. A limitation of Sato et al. [13] was that the stick figure animations did not provide an accurate reproduction of the dancer’s body and judges’ evaluations may differ between viewing stick figure animations and the actual dancers. An animation which retains a dancer’s body shape but anonymises the dancer’s identity is needed in order to evaluate dance performance based on kinematic criteria, and this approach may also be beneficial in hip-hop dance competition as an evaluation support tool.

Hopper and Sato [14] developed a new procedure for creating and retargeting humanoid animation skeletons onto anthropometrically scaled digital avatars. Humanoid animations such as from Hopper and Sato [14] with accurate movement representations may provide the means to investigate the unbiased judges’ evaluations of the movement elements in hip-hop dance performance. The purpose of this study was to determine the reliability of judges’ evaluations of hip-hop dance movements using identifiable and anonymous visualisation tools. More specifically, the study assessed the intra- and inter-rater reliability of hip-hop judges’ evaluations of performance, when observing anonymous humanoid animations, anonymous stick figure animations, and actual videos of hip-hop dance movements. We hypothesized that the humanoid animations would be associated with the highest intra- and inter-rater evaluation reliability of the three observation methods.

## Materials and methods

### Participants

Ten hip-hop dancers (six males and four females, age 21.6 ± 2.5 years, height 1.64 ± 0.06 m, weight 55.4 ± 5.8 kg, BMI 20.6 ± 1.6, 5.6 ± 5.2 years of hip-hop dance experience) and 10 judges participated in this study. Five of dancers were prize-winning dancers of national-level competitions and other five dancers had not received national accolades or were not competing at national level competitions. The judges (five male and five female) had 19.0 ± 4.0 years of hip-hop dance experience and 5.2 ± 2.6 years of experience as judges in hip-hop dance competitions. The Nagoya Gakuin University Research Ethics Committee approved the experimental procedure of the study, and written informed consent was obtained from all individual participants included in this study before the commencement of the experiment.

### Experimental procedures

Dancers were asked to perform ten cycles of the basic rhythmic movement by the down technique to a metronome pulse of 100 beats per minute [15], a fundamental skill in hip-hop dance in which the dancer repeatedly bounces the body by flexing and extending the trunk and lower extremities. The movements of the dancers were captured using an eight-camera motion capture system (Oxford Metrics, UK) sampled at 240 Hz. Two video cameras were positioned to the front and left of the dancers, sampling at 60 Hz. Spherical markers (10 mm diameter) were attached to the skin or clothing over 92 anatomical points, using the marker placement from Hopper and Sato [14].

### Data analysis

**Fig 1:**
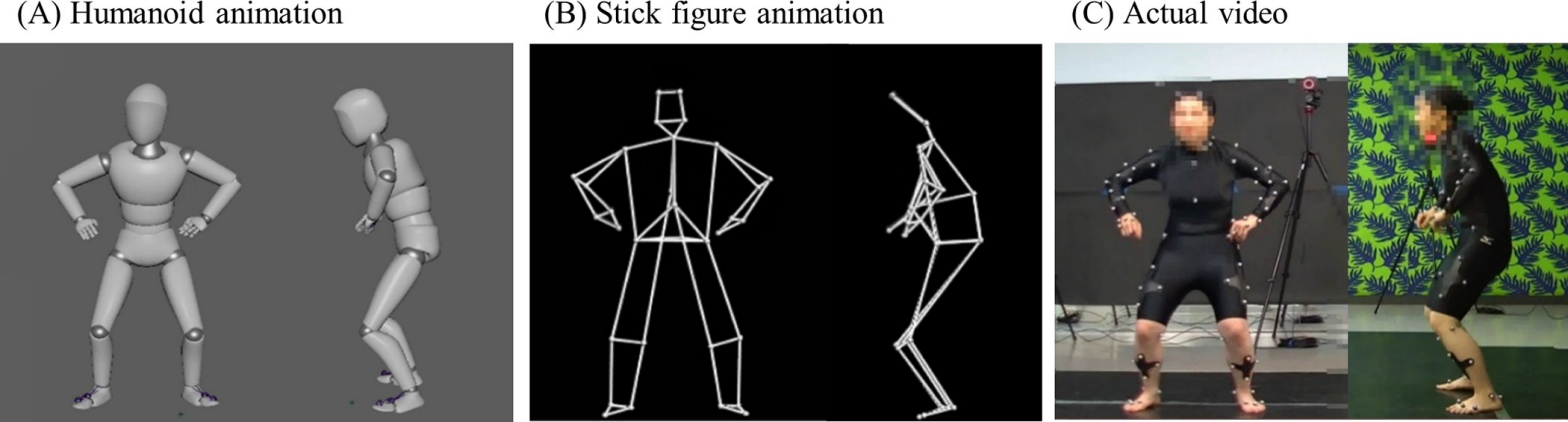
Examples of images of three observation methods. (A) Humanoid animation, (B) Stick figure animation, and (C) Actual video.

A humanoid animation was created as per Sato and Hopper [16] with 21 segments (Fig 1A). A stick figure animation that connected the markers with straight lines was created in Nexus motion capture software (Oxford Metrics, UK, Fig 1B). The number of segments and segment lengths in each animation were exactly the same because both animations were created using the same motion capture data. However, the thickness of each segment was set the same for all dancers in the humanoid animation as dancer girth and width anthropometric data was not measured [14].

The 10 judges evaluated the performance by observing the actual video, stick figure animation, and humanoid animation on a computer display. In each observation method, the frontal image and sagittal image were combined on a single screen using video editing software (EDIUS, JP). The image was adjusted so that the height of each dancer appeared the same. In the actual video, mosaic processing was performed only for the facial image (Fig 1C).

The judges were asked to evaluate 20 dance trials (i.e., 10 dancers multiplied by 2 observations for each) in random order during one observation method. Then they repeated this process using the other two observation methods. The order of the observation method was also set randomly between judges. The judges were not informed that they were repeating an evaluation of each dancer. The judges were instructed to grade each movement on a scale of 1–10, with 10 being the highest grade. They were able to declare their judgments up to a precision of two decimal places [17]. The scores were then standardised by conversion into T-scores.

### Statistics

The reliability of the evaluations was examined using inter- and intra-class correlation coefficients (ICC_2,1_ and ICC_1,1_, respectively [18]). Inter- and intra-class correlation coefficients higher than 0.80 represent very high reliability, and those between 0.60 and 0.79 represent moderately high reliability. The differences among the average values of the observation methods were examined by a one-way ANOVA. All statistical analyses were calculated using IBM SPSS Statistical software (v. 25.0, IBM, USA).

## Results

The scores of the humanoid animation, the stick figure animation, and the video were 5.61 ± 1.80, 5.57 ± 1.81, and 5.44± 1.64, respectively. There were no significant differences among observation methods.

The inter-class correlation coefficient for the evaluation of the humanoid animation demonstrated the highest reliability among the three methods (ICC_2,1_ = 0.929, 95% confidence intervals (CI) = 0.839–0.979, Table 2). Although the stick figure animation demonstrated moderate to high reliability (ICC_1,1_ = 0.781, 95% CI = 0.675–0.863), both the humanoid animation and the video demonstrated very high reliability in the intra-class correlation coefficient (ICC_1,1_ = 0.860, 95% CI = 0.792–0.905; and ICC_1,1_ = 0.880, 95% CI = 0.822–0.919, respectively).

**Table 2:** Intra- and inter-rater reliability for judges.

	Inter-rater reliability	Intra-rater reliability										
		Judge A	Judge B	Judge C	Judge D	Judge E	Judge F	Judge G	Judge H	Judge I	Judge J	all Judges
Stick figure animation	0.860	0.848	0.890	0.905	0.798	0.885	0.614	0.777	0.770	0.816	0.617	0.781
Humanoid animation	0.929	0.943	0.918	0.924	0.896	0.787	0.902	0.791	0.855	0.861	0.805	0.860
Video	0.877	0.881	0.972	0.905	0.802	0.857	0.785	0.919	0.948	0.961	0.765	0.880

## Discussion

This study examined the reliability of judges’ evaluations of hip-hop dance movements visualised through a humanoid animation, a stick figure animation, and an actual video of hip-hop dance movement. Our hypothesis was supported that the intra- and inter-rater reliability of the humanoid animation demonstrated the highest reliability among the three methods. In this study, the humanoid animation for each dancer was created using the same digital avatar, which was adjusted to the anthropometric dimensions of each dancer. In addition, the judges evaluated the image, which was adjusted so that the height of each dancer appeared the same. Therefore, the humanoid animation was able to minimise the judges’ potential evaluation biases of the dancers’ movements stemming from clothes, facial attractiveness, physical features, shapes, and gender difference.

Although there was little difference between the video and the humanoid animation in the intra-rater reliability, the inter-rater reliability of the video was lower than that of the humanoid animation. In the video, the image of the height was adjusted so that the height of each dancer appeared the same and mosaic processing was also executed to include only the dancer’s facial image. However, the judges were able to recognise the body shape and gender of the dancer. Kondo et al. [19] investigated the influence of gender membership in the judgement of face attractiveness and reported that this judgment might be performed using different frames for male and female faces. Fan et al. [20] reported that the body volume divided by the square of the height was related to perceptions of female physical attractiveness. The factors relating to the difference in gender and body shape might have affected the inter-rater reliability of hip-hop performance in the video as opposed to the humanoid animation.

In the stick figure animation and the humanoid animation, variations in dancers’ facial features, physical features, body shapes, and gender difference were minimised. Although the dancers’ movements were consistent between the two visualisation methods, the intra- and inter-rater reliability of the stick figure animation demonstrated the lowest evaluation reliability among the three methods. This result was in agreement with previous study Sato et al. [13]. People can distinguish sex and identify individual persons only observing small number of dots or stick figure animation representing a walking person [10, 21]. From these previous studies, it is assumed that people are able to gain much perceptual information from stick figure animation, on the other hand, gender difference bias which was detected from stick figure animation might affect the reliability of the stick figure animation. In this study, judges observed both frontal and sagittal perspectives of the stick figure animations. In hip-hop dance competitions, although judges usually sit in front of the stage to evaluate performance, judges evaluate dancers’ performance from various directions because dancers perform facing various directions. As the stick figure animation was created using only straight lines, the judges may perceive the movement as being two-dimensional and far from the typical perspective of dancers in competition. However, it is still unclear why stick figure animations did not provide sufficient information to evaluate dance movement. Further studies should be undertaken to investigate about visual cues related to not only to kinematic pattern, but also force or expression.

In the 2018 Youth Olympic Games, six interlinked categories were used to evaluate hip-hop dancer performances; 1) Technique, 2) Performativity, 3) Creativity, 4) Variety, 5) Musicality, and 6) Personality [2]. Our results suggest that judges may have difficulty in evaluating dance performance through discrete criteria and interdependences likely exist between the criteria used in the 2018 Youth Olympic Games. Future research is required to assess the relationship among the six categories used for hip-hop evaluation with the aim of scrutinising and developing a reliable evaluation system.

In artistic gymnastics, judges provide a “Difficulty score” and the “Execution Score” [4]. In figure skating, the “Technical Element Score” and the “Program Component Score” are used to evaluate a performance [3]. Judging scores have been reported to have high reliability for these artistic sporting competitions [4, 6, 22]. In hip-hop dance, the evaluation could be divided into technical and expression criteria. Our results suggested that the kinematic components of technical criteria could be developed by judges observing humanoid animations representing dancers' movements. In addition, the humanoid animation could be used as an evaluation support tool in hip-hop dance competitions. Other than the judging system, there are numerous objective and subjective factors which affect to the reliability of judging (e.g., the number of competitors in a session the different variability of scores, judges’ seat positions and view angle to the performer and the judge’s experience, [4]). Using artistic sports competitions as a benchmark, the reliability of hip-hop judging scores need further assessment and the judging categories should be further considered in the interests of developing reliable judging system as hip-hop becomes an internationally recognised sporting competition.

A few limitations were associated with this study. First, it should be noted that the sample size was small [23]. Second, in the humanoid animation, the length of the segment was adjusted to an actual dancer, but the thickness of segment was not. An animation with actual dancer’s body shape which is anonymised confounding aesthetic factors should be developed. Third, the judges might identify individual dancers when they repeated observing same dancer within and between observation methods although they were not informed that they were repeating an evaluation. Further studies should be undertaken to investigate these issues.
